# HER2 discordance between primary and metastatic gastric cancer: a systematic review and meta-analysis

**DOI:** 10.1007/s12094-026-04359-9

**Published:** 2026-05-12

**Authors:** Francisco Cezar Aquino Moraes, Luis Henrique Rios Moreira Rego, Thiago Gorayeb Rebelo, Gustavo Tadeu Freitas Uchôa Matheus, Ana C. Melo

**Affiliations:** 1 Department of Morphology and Genetics, Federal University of São Paulo, São Paulo, São Paulo Brazil; 2 State University of Piauí, Teresina, Piauí Brazil; 3 University of Ottawa, Ottawa, ON Canada; 4 Federal University of Triângulo Mineiro, Uberaba, Minas Gerais Brazil; 5 Department of Pathology and Genomic Medicine, Houston Methodist Hospital, Houston, TX USA

**Keywords:** Gastric cancer, HER2 expression, Metastasis, Meta-analysis

## Abstract

**Purpose:**

Gastric cancer (GC) remains a major global health burden, with poor survival rates. HER2 is a key biomarker for targeted therapy, but discordance between primary tumors and metastases may impact treatment decisions. This meta-analysis evaluated the prevalence and clinical relevance of HER2 status differences between primary gastric cancers and metastatic lesions.

**Methods:**

A systematic search of PubMed, Embase, and Cochrane was conducted for studies assessing HER2 status in matched primary and metastatic gastric cancer samples. Pooled proportions and 95% confidence intervals (CIs) were calculated using random-effects models. Subgroup analyses by metastatic site were performed. Statistical analyses were conducted using R software, version 4.2.3.

**Results:**

This meta-analysis included 3,515 patients across 20 studies. The pooled proportion of HER2-positive expression in primary gastric tumors was 13% (95% CI: 12–15%; I^2^ = 68.7%), while HER2-negative tumors accounted for 74% (95% CI: 72%-76%; I^2^ = 94.9%). In metastatic sites, the overall pooled proportion of HER2-positive lesions was 18% (95% CI: 16%-20%; I^2^ = 77.3%), with notable variation across anatomical locations: 38% in lung metastases, 31% in liver metastases, 19% in lymph nodes, and 7% in peritoneum. Conversely, HER2-negative metastases accounted for 82% overall (95% CI: 80%-84%; I^2^ = 78.2%), with proportions of 93% in peritoneum, 81% lymph nodes, 69% in liver metastases, and 62% in lung.

**Conclusions:**

Significant heterogeneity in HER2 expression between primary and metastatic gastric cancer underscores the need for reassessment of HER2 status in metastatic sites. Relying solely on primary tumor samples may lead to underestimation of HER2 positivity and suboptimal therapeutic decisions. Incorporating site-specific HER2 testing into clinical practice may enhance patient selection for HER2-targeted therapies and improve treatment outcomes.

**Graphic Abstract:**

**Figure:**
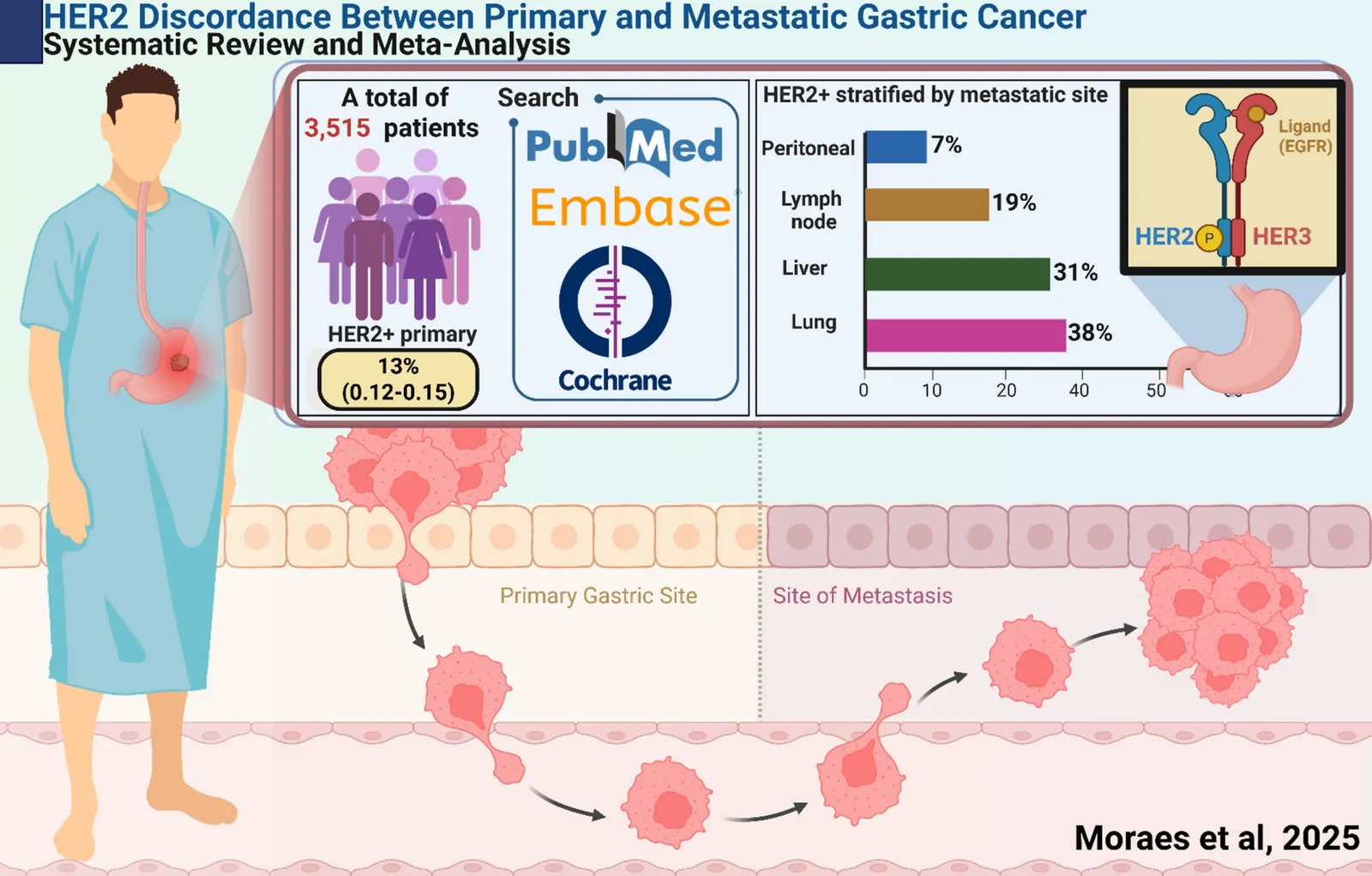

**Supplementary Information:**

The online version contains supplementary material available at https://doi.org/10.1007/s12094-026-04359-9.

## Introduction

Gastric adenocarcinoma, or gastric cancer (GC), is the fifth most common tumor worldwide [1, 2]. Along with gastroesophageal junction (GEJ) adenocarcinomas, advanced or metastatic cases still present a grim prognosis [3]. The five-year survival rate is approximately 20% for stage II and III, and the median overall survival (OS) for patients treated with standard platinum-fluoropyrimidine–based cytotoxic chemotherapy is around 10 months for patients in stage IV [4–7]. Recently, targeted therapies against emerging biomarkers have improved survival outcomes for patients with GC/GEJ [8–10]. In particular, the human epidermal growth factor receptor 2 (HER2) has become a well-established therapeutic target in this setting [11, 12]. The addition of trastuzumab, an anti-HER2 monoclonal antibody, to cytotoxic chemotherapy was evaluated in the phase III ToGA trial for patients with unresectable or metastatic HER2-positive GC/GEJ, demonstrating a significant increase in median OS (13.8 vs. 11.1 months; *p* = 0.0046) [13]. As a result, the combination of anti-HER2 therapy and chemotherapy has become the standard treatment for these patients.

The HER2 protein, encoded by the ERBB2 oncogene, is one of the most well-studied aspects of advanced-stage gastric cancer (GC) [14]. HER2 can form heterodimers with other members of the family, including EGFR, HER3, or HER4, which activate the intracellular autophosphorylation process of the tyrosine kinase domain [15–17]. This results in increased HER2 activation, which stimulates cellular signaling transduction through PI3K, AKT, RAS, and RAF, consequently promoting cancer progression [18]. HER2 overexpression in GC confers a malignant phenotype to the tumor, as it promotes cell proliferation and survival through the regulation of key cell cycle proteins such as SKP2 and p27/cdk2 [19, 20]. Additionally, HER2 overexpression is directly proportional to the increase in vascular endothelial growth factor (VEGF), which enhances angiogenesis, significantly increasing tumor growth and metastasis [21, 22]. Furthermore, HER2 activation can enhance the migratory and invasive capacity of neoplastic cells due to the activation of the Wnt/β-catenin pathway, which triggers epithelial-mesenchymal transition (EMT) [23, 24].

The overexpression of the HER2 protein or HER2 gene amplification (HER2 positivity) is found in approximately 22% of all GC/GEJ cases [13, 25–27]. HER2 status can be determined by assessing expression levels through immunohistochemistry (IHC) or by identifying HER2 gene amplification using in situ hybridization (ISH) [28]. Both breast and GC/GEJ tumors may exhibit incomplete membrane staining, but in GC/GEJ the pattern is often lateral or basolateral and is interpreted differently, and substantial intratumoral heterogeneity in HER2 expression is more common [26]. This biology contributes to greater variability in HER2 status between primary and metastatic sites in GC/GEJ, increasing the risk of false-negative classification and potential undertreatment [26]. In contrast, breast cancer shows a relatively low rate of discordance between primary tumors and corresponding metastases (approximately 5.5%), in part reflecting more uniform staining and standardized interpretation [29–33]. Nevertheless, discrepancies in HER2 positivity—whether synchronous or metachronous—remain a significant challenge for identifying patients who may benefit from HER2-targeted therapies [11, 30–33]. Thus, to better characterize and quantify these discordances, we conducted a comprehensive systematic review and meta-analysis of the existing literature.

## Methods

### Protocol and registration

We conducted our meta-analysis systematically in accordance with the guidelines outlined in the Cochrane Handbook for Systematic Reviews of Interventions. The findings were reported following the PRISMA (Preferred Reporting Items for Systematic Reviews and Meta-Analyses) guidelines [34]. The final protocol was registered with PROSPERO under the number CRD42025650040, prior to the commencement of the study in February 2025.

### Eligibility criteria

We included studies that met the following eligibility criteria: (1) participants had primary gastric cancer with metastasis; (2) studies assessed HER2 status in both the primary tumor and metastatic sites; and (3) studies reported HER2 discordance between primary gastric cancer and metastases. Studies with overlapping populations, as well as reviews, opinion articles, technical reports, guidelines, animal studies, and in vitro experiments, were excluded. Additionally, only articles published in English were considered and no restrictions on the publication dates of included studies were applied.

### Search strategy

We conducted a systematic and comprehensive search of published studies across several major databases, including PubMed, Embase, and the Cochrane Library. Our search extend beyond full-text articles to include abstracts and scientific presentations, ensuring a thorough review of the available literature. To optimize retrieval, we customized the Medical Subject Headings (MeSH) and search terms according to the syntax requirements of each database, using Boolean operators (OR, AND) to combine terms effectively. To further broaden our pool of relevant studies, we carefully reviewed the reference lists of all included articles and of related systematic reviews. Additionally, alert systems were configured in each database to notify us of any new publications matching our predefined criteria. The search strategy was executed collaboratively by two authors (F.C.A.M and L.H.R.M.R), who conducted the database searches and screened references from the identified articles. This comprehensive approach was designed to maximize the inclusion of relevant evidence.

### Data extraction and risk of bias assessment

To summarize the main findings, two authors (G.T.F.U.M. and T.G.R.) extracted the data from the included articles, including the authors and publication year, study design, patient sample characteristics (size, age, clinical and pathological data, and study group), assessment method, and conclusions regarding HER2 discordance between the primary tumor and metastasis.

Risk of bias was assessed using the Newcastle–Ottawa Scale (NOS) [35], which evaluates the quality of non-randomized studies across three domains: selection of study groups, comparability of groups, and ascertainment of outcome. Each study could receive a maximum of nine stars. Studies scoring 8–9 stars were considered high quality (low risk of bias), whereas those scoring 7 stars were classified as having a moderate risk of bias. The risk of bias was independently assessed by two reviewers (F.C.A.M. and G.T.F.U.M.), and any discrepancies were resolved with the assistance of a third reviewer, who made the final decision regarding study inclusion in the analysis.

To evaluate publication bias in this single-arm meta-analysis, we used the Doi plot in conjunction with the LFK index, considering values outside the ± 1 range as indicative of significant asymmetry [36].

### Endpoints and definitions

The primary outcome of interest was the rate of HER2 discordance between primary gastric cancer and metastatic lesions. The secondary outcomes were: (1) HER2 negative expression in metastases; (2) HER2 positive expression in metastases; (3) HER2 negative expression in primary tumors; (4) HER2 status assessment in primary tumors. HER2 is a critical biomarker for targeted therapies and may, in some cases, be overexpressed in both primary tumors and metastases. HER2 discordance refers to the difference in HER2 status between the primary tumor and its metastases, which can have a direct impact on treatment strategies.

### Statistical analysis

We conducted a single-arm meta-analysis using the 'meta' package in R(version 4.3.2) [37]. The proportions reported in the included studies were logit-transformed, and study weights in the pooled analysis were estimated using the inverse-variance method. The degree of heterogeneity was assessed using the Tau^2^ statistic, which quantifies the variance in true effect sizes, and the I^2^ statistic, which measures the percentage of variation attributable to heterogeneity. Heterogeneity was considered statistically significant when p < 0.10 or I^2^ > 25% [38]. To explore the influence of individual studies on overall heterogeneity, a leave-one-out sensitivity analysis was performed. In addition, subgroup analyses were conducted according to the site of metastasis. A random-effects model was applied to all outcomes.

## Results

**Fig. 1:**
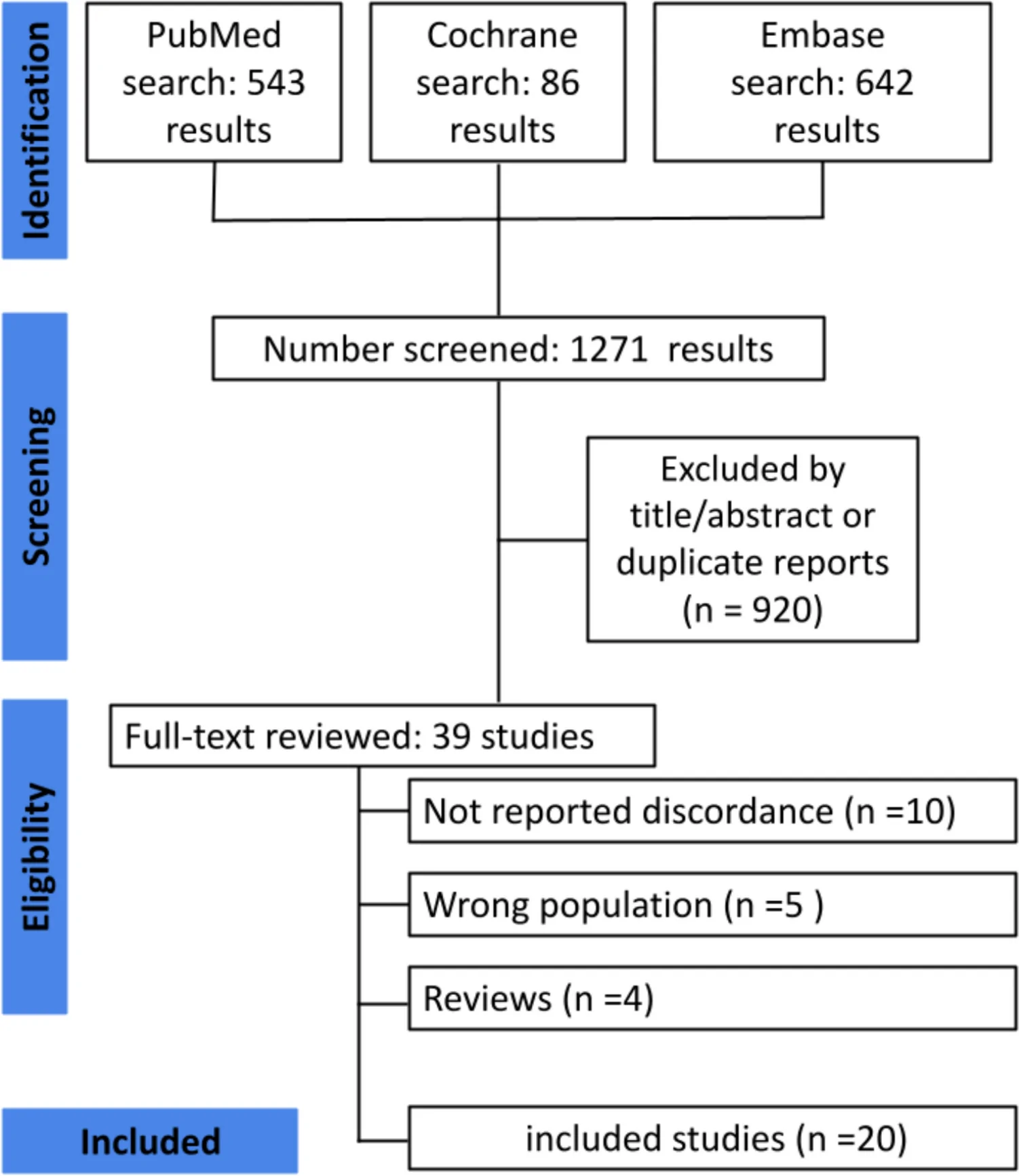
Preferred Reporting Items for Systematic Review and Meta-Analysis (PRISMA) flow diagram of study screening and selection

The initial search identified 1,271 articles from three databases (PubMed, Embase, and Cochrane). After deduplication, 365 duplicates were removed, leaving 906 records for title and abstract screening. Of these, 39 studies were deemed potentially eligible and assessed through full-text review. Among them, 10 were excluded due to not reporting discordance, 5 report wrong population and 4 were reviews. Finally, 20 studies were included in this meta-analysis [26, 30, 31, 33, 39–54] (Fig. 1).

### Study characteristics

A total of 3,515 patients, with 3,537 samples were included across the 20 studies selected for this meta-analysis. With sample sizes ranging from 35 to 575 patients. HER2 assessment was performed using immunohistochemistry (IHC) in all studies, with fluorescence in situ hybridization (FISH) used as a confirmatory method in some cases. Among the overall population, 258 samples were classified as HER2-positive in the primary tumor, while HER2-positive expression in metastatic lesions was observed in 263 samples. Additionally, 1,394 metastatic cases were HER2-negative, with 1,622 samples being classified as HER2-negative in primary tumor (Table 1).

**Table 1:** Design and characteristics of studies included in the Meta-analysis

Study	Country	Number of patients	Method of detection of HER2	HER2 + primary site (n)	HER2—primary site (n)	HER2 + metastasis (n)	HER2—metastasis (n)	NOS
Amato, 2017[39]	Italy	41	IHC/FISH	6	35	10	31	7
Bozzetti, 2011[31]	Italy	72	IHC/FISH	5	34	7	32	8
Cho, 2013[33]	South Korea	498	IHC/SISH	17	80	16	81	7
Fassan, 2012[41]	Italy	47	IHC/CISH	12	34	12	34	9
Fusco, 2013[42]	Italy	95	IHC/FISH	17	129	10	136	8
Geng, 2013[40]	China	206	IHC	21	89	24	86	7
Gumusay, 2015[43]	Turkey	74	IHC/SISH	5	69	4	70	9
Huemer, 2020[44]	Austria	183	IHC/ISH	37	75	45	149	8
Imano, 2012[45]	Japan	35	IHC/FISH	1	17	1	34	8
Kim, 2011[46]	Korea	575	IHC/FISH	12	210	25	197	9
Kochi, 2013[47]	Japan	102	IHC/FISH	25	77	22	80	9
Jung, 2013[48]	Brazil	118	IHC	1	62	1	35	9
Kolbe, 2022[26]	Germany	548	IHC/ISH	6	NA	9	NA	8
Marx, 2009[49]	Germany	166	IHC/FISH	27	139	11	58	8
Park, 2015[30]	South Korea	358	IHC	16	167	10	165	8
Qiu, 2017[50]	NA	116	IHC/FISH	5	111	4	40	9
Selcukbiricik, 2014[51]	Turkey	81	IHC/SISH	17	64	21	60	7
Shibata, 2014[52]	Japan	37	IHC/ FISH	6	31	7	42	8
Tsapralis, 2012[53]	Greece	120	IHC/CISH	19	101	19	26	7
Wong, 2015[54]	NA	43	IHC/SISH	3	40	5	38	9

IHC, Immunohistochemistry; FISH, Fluorescence In Situ Hybridization; SISH, Silver In Situ Hybridization; CISH, Chromogenic In Situ Hybridization; ISH, In Situ Hybridization; NA, Not Available; NOS, Newcastle–Ottawa Scale

### HER2 status in primary gastric cancer

**Fig. 2:**
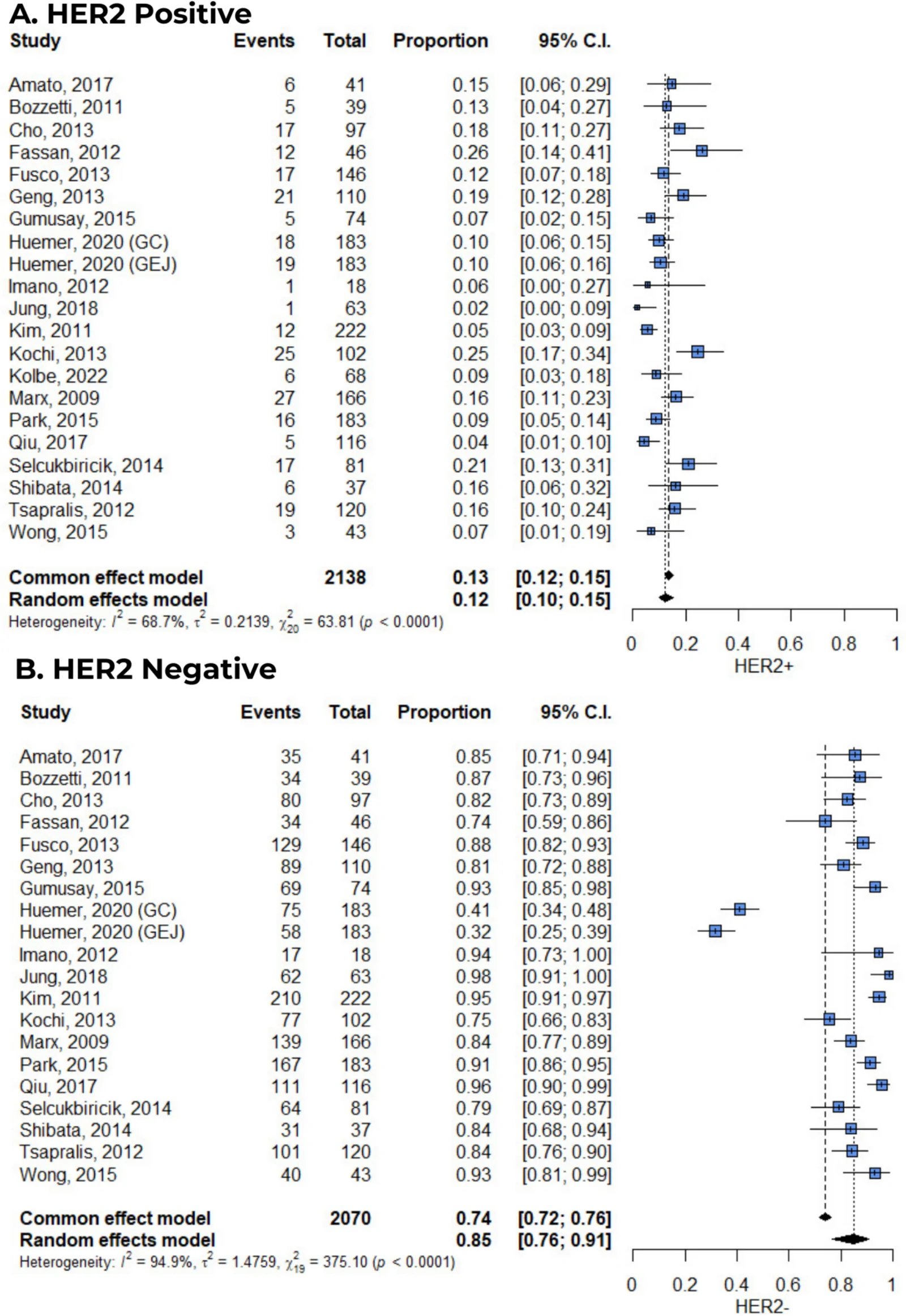
Pooled proportion of HER2 expression in primary gastric cancer. **A** Forest plot showing the proportion of HER2-positive tumors in primary gastric cancer across included studies. **B** Forest plot showing the proportion of HER2-negative tumors in primary gastric cancer

The pooled proportion of HER2-positive tumors in primary gastric cancer was 0.13 (95% CI: 0.12–0.15; I^2^ = 68.7%), based on 258 HER2-positive cases among 2,138 samples. For HER2-negative tumors, the pooled proportion was 0.74 (95% CI: 0.72–0.76; I^2^ = 94.9%), comprising 1,622 HER2-negative cases out of 2,070 samples with primary gastric cancer, as shown in Fig. 2.

### HER2 status in metastatic gastric cancer

**Fig. 3:**
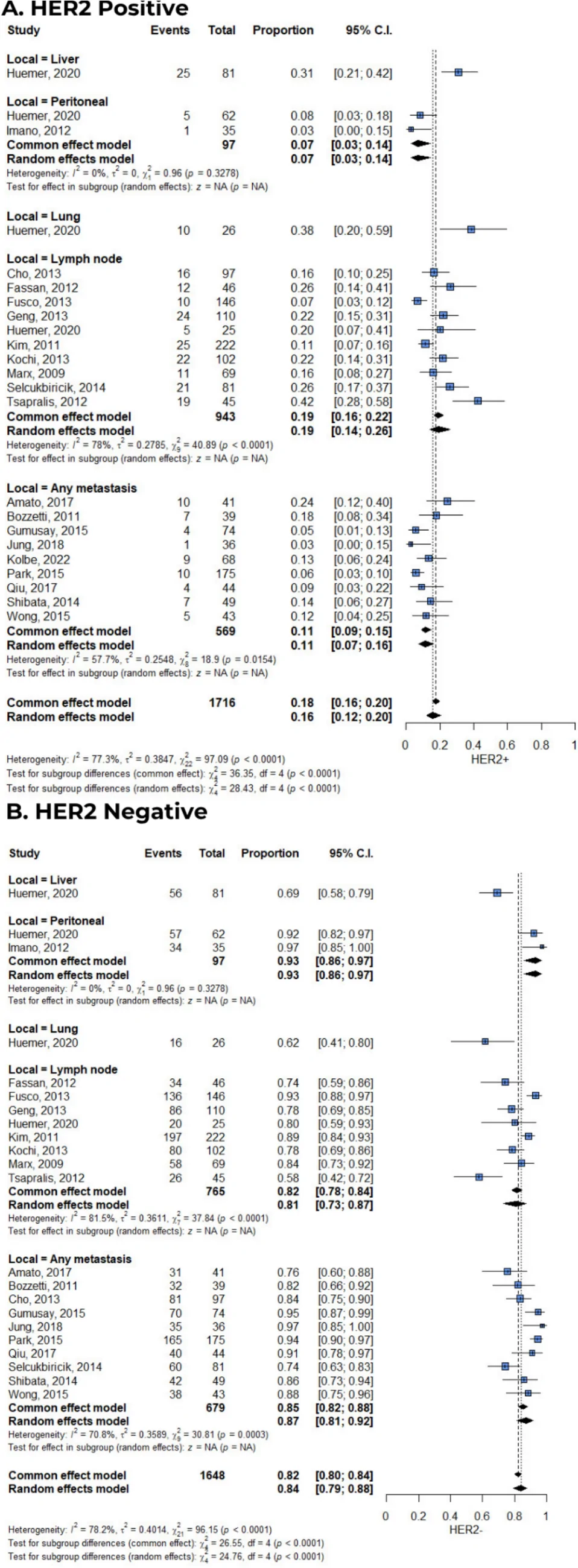
Pooled proportion of HER2 expression in metastatic gastric cancer. **A** Forest plot showing the proportion of HER2-positive cases among patients with metastatic gastric cancer, stratified by metastatic site. **B** Forest plot showing the proportion of HER2-negative cases among patients with metastatic gastric cancer, also stratified by metastatic site

The pooled proportions of HER2 status in metastatic gastric cancer, stratified by metastatic site, are shown in Fig. 3. In Fig. 3A, the pooled proportion of HER2-positive metastatic lesions was 0.18 (95% CI: 0.16–0.20; I^2^ = 77.3%), based on 263 HER2-positive metastases among 1,716 samples. Subgroup analyses using random-effects models demonstrated HER2 positivity rates of 0.07 (95% CI: 0.03–0.14) in peritoneal metastases, 0.38 (95% CI: 0.20–0.59) in lung metastases (one study), 0.31 (95% CI: 0.21–0.42) for liver metastases (one study), 0.19 (95% CI: 0.14–0.26) in lymph node metastases, and 0.11 (95% CI: 0.07–0.16) in studies with unspecified metastatic site.

In Fig. 3B, the pooled proportion of HER2-negative metastatic lesions was 0.82 (95% CI: 0.80–0.84; I^2^ = 78.2%), based on 1,394 HER2-negative metastases among 1,648 samples. Stratified analyses by random-effects models showed HER2 negativity rates of 0.69 (95% CI: 0.58–0.79) in liver metastases (one study), 0.93 (95% CI: 0.86–0.97) in peritoneal metastases, 0.62 (95% CI: 0.41–0.80) in lung metastases, 0.81 (95% CI: 0.73–0.87) in lymph node metastases and 0.87 (95% CI: 0.81–0.92) in studies without metastatic site specification.

### Quality assessment

Based on the Newcastle–Ottawa Scale (NOS), we assessed the risk of bias across the included studies. Fifteen studies were rated as having a low risk of bias, of which eight received 8 stars and seven achieved the maximum score of 9 stars. The remaining five studies were classified as having a moderate risk of bias, each receiving 7 stars. Most of these studies lost points in the domains "Representativeness of the exposed cohort" and "Assessment of outcome." A detailed breakdown of the NOS assessment for each study is provided in Supplementary Table S4.

**Fig. 4:**
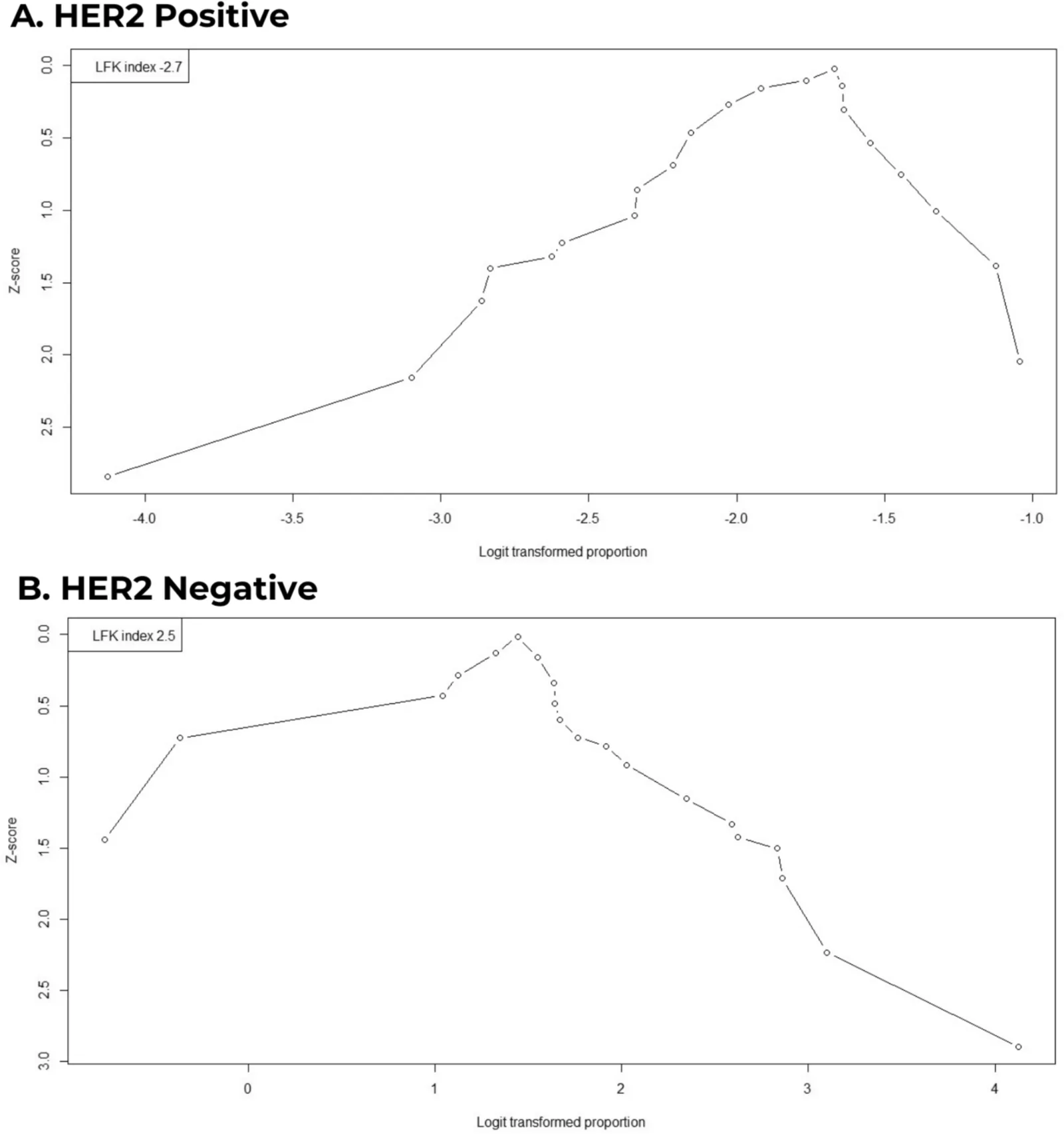
Doi plot analysis for the HER2 status in primary gastric cancer. **A** HER2 positive and **B** HER2 negative

To assess publication bias, Doi plots were generated and LFK index was calculated for each analysis. For HER2-positive primary gastric cancer, the LFK index was -2.7, indicating evidence of publication bias (Fig. 4A). Similarly, for HER2-negative primary tumors, the LFK index was 2.5, suggesting major asymmetry and potential publication bias (Fig. 4B). Regarding metastatic gastric cancer, the LFK index for HER2-positive cases was -2.42, indicating asymmetry, while the index for HER2-negative cases was 2.42, consistent with significant publication bias. The corresponding Doi plots for metastatic tumors are shown in Supplementary Figures S1A and 1B.

### Sensitivity analysis

A leave-one-out sensitivity analysis was performed for all outcomes. The omission of individual studies did not result in any substantial reduction in heterogeneity across the analyses. The corresponding leave-one-out plots are presented in Supplementary Figure S2.

## Discussion

In this systematic review and meta-analysis, we evaluated HER2 expression using immunohistochemistry and fluorescence in situ hybridization (FISH) in 258 HER2-positive and 1,622 HER2-negative primary gastric cancer samples drawn from 20 studies. Among the corresponding metastatic sites, there were 263 HER2-positive and 1,394 HER2-negative specimens. Our results showed that HER2 positivity in gastric cancer is 13% (95% CI: 0.12–0.15), increasing to 18% (95% CI: 0.16–0.20) in metastatic lesions.

Approximately 22% of gastric and gastroesophageal junction (GEJ) cancers exhibit HER2 overexpression or gene amplification, identifying a subgroup for whom personalized therapy is particularly relevant [11]. In 2010, the ToGA trial established trastuzumab—a monoclonal antibody targeting HER2—as a first-line treatment for patients with HER2-positive gastric adenocarcinoma. In this phase III randomized trial involving 594 patients, the addition of trastuzumab significantly improved median overall survival (mOS 13.8 vs. 11.1 months; *P* = 0.0048), median progression-free survival (mPFS 6.7 vs. 5.5 months; *P* = 0.0002), and objective response rate (47% vs. 35%; *P* = 0.0017) [13]. Consequently, trastuzumab was approved as the standard of care for HER2-positive gastric cancer, typically in combination with capecitabine or 5-fluorouracil plus cisplatin, and has since been adopted worldwide, including in the United States, China, the United Kingdom and Japan [55].

Accurate assessment of HER2 status—using immunohistochemistry (IHC) or in situ hybridization (ISH)—is essential before initiang anti-HER2 therapy in oncology practice [56]. Accordingly, numerous guidelines recommend routine HER2 testing for patients with locally advanced, recurrent, or metastatic unresectable gastric or GEJ adenocarcinoma [30, 33, 57]. However, because gastric cancers often display marked intertumoral and intratumoral heterogeneity, false-negative results can occur [14, 15, 27, 58]. Therefore, repeat HER2 testing is strongly recommended on surgically resected specimens when the initial endoscopic biopsy is negative. In inoperable advanced disease, endoscopic biopsy remains the only available tissue source for HER2 evaluation; however, obtaining additional biopsies carries procedural risks due to the invasive nature of the procedure. An alternative approach is the analysis of malignant effusions—such as ascitic or pleural fluid—which can be collected via minimally invasive procedures and performed repeatedly, offering a valuable means of assessing HER2 expression in metastatic cell clones.

Owing to the aggressive nature of gastric cancer, its nonspecific clinical presentation, and the lack of robust, large-scale diagnostic protocols in Western populations, many patients are diagnosed at an advanced stage, with up to 40% exhibiting malignant ascites or peritoneal metastases [59]. The cell block technique enables cytological specimens to be processed as formalin-fixed paraffin-embedded tissue for molecular analyses and IHC [60, 61]. Indeed, Wong et al. [62] evaluated HER2 status concordance in 46 malignant effusion cell blocks derived from gastric adenocarcinoma and reported 100% agreement with paired histological specimens. Although these results support the feasability of parallel HER2 testing across both sample types, only 39% of the cytological specimens in that study were paired with corresponding histological blocks for concordance analysis [62]. In contrast, our pooled analysis indicate that HER2-positive status appears to be lower in the primary gastric tumors 13%(95% CI: 0.12–0.15), but rises to 18% (95% CI: 0.16–0.20) in metastatic lesions. This discrepancy likely reflects intratumoral heterogeneity and the selective outgrowth of HER2-positive clones, which possess a relatively greater invasive potential and therapeutic resistance, thereby explaining their increased prevalence at distant metastatic sites under the pressure of cancer-driven natural selection.

When site-specific HER2 expression was examined across metastatic lesions, positivity was most frequent in lung metastases (38%; 95% CI: 0.20–0.59), followed by liver metastases (31%; 95% CI: 0.21–0.42), lymph node metastases (19%; 95% CI: 0.16–0.22), metastases at any site (11%; 95% CI: 0.09–0.15), and peritoneal metastases** (**7%; 95% CI: 0.03–0.14). At first glance, the rate of HER2 positivity in lymph node metastases appears higher than in the primary tumor (19% vs. 13%). However, several factors must be considered. Even with increasingly standardized HER2 assessment methods, approximately 5% of cases exhibit conversion from negative to positive and 18% from positive to negative, reflecting not only intratumoral heterogeneity but also potential sampling bias. HER2 testing in gastric cancer is deemed reliable when at least four to five endoscopic biopsies are obtained from the gastric mucosa [63, 64]. By extension, a similar sampling standard could theoretically enhance biomarker validation in metastatic sites, although such extensive tissue collection is often impractical in clinical settings.

Although our meta-analysis offers meaningful insights into the differential frequency of HER2 positivity between primary and metastatic gastric cancer sites, several limitations should be recognized. First, data on treatment response—particularly to anti-HER2 therapies—were unavailable, precluding assessment of therapeutic efficacy at both primary and metastatic sites. Second, information on other relevant biomarkers, including genomic landscape features, Helicobacter pylori infection status, microsatellite instability (MSI) status, and PD-1 expression, was often missing. This limitation not only constrains the generalizability of our findings but also highlights the need for further research to elucidate specific associations with key gastric cancer factors. Accordingly, future investigations—ideally prospective studies incorporating multiple biomarkers and minimally invasive tools such as circulating tumor DNA (ctDNA), alongside targeted therapy application—are warranted to deepen our understanding of the prognostic impact of HER2 discordance in gastric cancer.

## Conclusions

Our meta-analysis revealed that HER2 status may exhibit greater discordance than previously observed between primary gastric tumors and their corresponding metastases. The higher rate of HER2 positivity in metastatic lesions underscores not only the dynamic nature of tumor biology—where cellular clones undergo natural selection during the metastatic process—but also highlights potential limitations of classifications based solely on primary tumor biopsies. This heterogeneity raises therapeutic concerns, as patients who might benefit from anti-HER2 therapies may be underrepresented due to the molecular status of the metastatic site. Therefore, we recommend that HER2 status be reassessed whenever feasible, and that such findings be integrated with molecular data obtained through minimally invasive technologies, such as circulating tumor DNA (ctDNA).

## Supplementary Information

Below is the link to the electronic supplementary material.

Supplementary file1 (DOCX 213 KB)

## Data Availability

All data generated or analyzed during this study are included in this published article [and its supplementary information files].
